# Commentary: Midbrain projection to the basolateral amygdala encodes anxiety-like but not depression-like behaviors

**DOI:** 10.3389/fnmol.2023.1117121

**Published:** 2023-02-08

**Authors:** Jihu Zhao, Peng Sun, Heng Liu

**Affiliations:** ^1^Department of Medicine, Qingdao University, Qingdao, Shandong, China; ^2^Department of Neurosurgery, The Affiliated Hospital of Qingdao University, Qingdao, Shandong, China

**Keywords:** anxiety disorders, midbrain, ventral tegmental area, basolateral amygdala, anxiolytic

## Introduction

Anxiety disorders are common chronic mental illnesses with complex causes, a high relapse rate, and a high prevalence of depressive symptoms (Zwanzger, 2016). Anxiety disorders are characterized by panic, nervousness, and irritability, as well as somatic and behavioral symptoms (Nechita et al., 2018). Anxiolytics are frequently used in conjunction with antidepressants in clinical practice to treat anxiety disorders. Despite the fact that the therapeutic effect is satisfactory, the drug combination causes uncontrollable side effects (Behlke et al., 2020). As a result, 273 million people worldwide suffer psychological and physical pain as a result of anxiety disorders (Whiteford et al., 2013). It was recently discovered that the midbrain dopamine system is a critical structure that regulates mood, motivation, reward, and salience. Furthermore, anxiety and depression are linked to the midbrain dopamine system (Mitsi and Zachariou, 2016). In 2013, Chaudhury et al. (2013) discovered that the ventral tegmental area (VTA) of the midbrain led to a sub-circulation of dopaminergic neurons in the nucleus accumbens (NAc) that rapidly modulated depression-related behavior. Similarly, VTA can be projected onto the basolateral amygdala (BLA), a critical part of the human brain that regulates anxiety processes (Felix-Ortiz et al., 2013). However, the neural circuitry mechanisms in anxiety disorders and anxious depressive states remain unknown. Furthermore, the relationship between VTA-BLA dopamine neurons and anxiety disorders requires additional research.

## The decreasing activity of VTA project to BLA neurons induces the anxiety-like behavior

**Figure 1:**
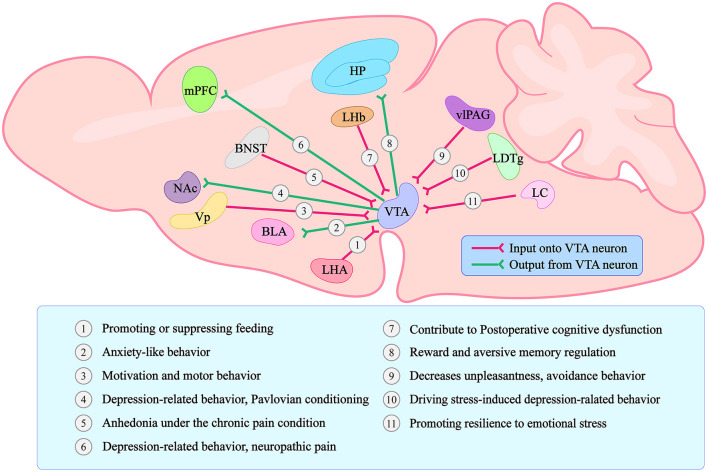
Graphical summary of the VTA connections with other brain regions and the main functions regarding each different projection. Forms and positions can only represent the general structure and position. The lines with arrows only represent a simplified schematic of the general projection pattern. VTA stands for Ventral Tegmental Area; LHA stands for lateral hypothalamic area; BLA stands for basolateral amygdala; Vp stands for ventral pallidum; NAc stands for Nucleus Accumbens; BNST stands for bed nucleus of the stria terminalis; mPFC stands for medial prefrontal Cortex; LHb stands for lateral habenula; HP stands for Hippocampus; vlPAG stands for ventrolateral periaqueductal gray; LDTg stands for laterodorsal tegmentum; LC stands for locus coeruleus.

Because anxiety and depression frequently coexist, the line between these two disorders has always been blurred (Demyttenaere and Heirman, 2020). We learned from the World Health Organization's (WHO) International Classification of Diseases (ICD) that symptoms of anxiety and depression frequently overlap. Anxiety disorders can also cause depressed moods and loss of interest, and depression can cause anxiety. However, the two diseases have distinct typical symptoms (Fang et al., 2019; Williamson et al., 2021). Unconditioned panic attacks, hyperarousal, and compulsions can occur in patients with anxiety disorders. Depression patients exhibit core symptoms such as a lack of pleasure, negative psychology, and emotion. As the midbrain limbic dopamine system has been studied further in recent years, researchers have discovered that the amygdala receives DA neuronal projections from the VTA and is involved in the emergence of anxiety-like behavior (Nguyen et al., 2021). Under optogenetic conditions, VTA-BLA and CeA-VTA DA neurons successfully modulate anxiety-like behavior in mice (Jiang et al., 2021). Furthermore, depression-like behavior in mice is regulated by VTA-PFC, VTA-NAC, LDTg-VTA, and LC-VTA DA neurons (Saddoris et al., 2015; Isingrini et al., 2016; Fernandez et al., 2018; Huang et al., 2020). A VTA is a complex brain structure made up of 60% dopaminergic neurons (DA neurons), 35% GABAergic neurons (GABA neurons), and 5% glutamatergic neurons (Yamaguchi et al., 2015). It regulates the release of neurotransmitters and peptides, which controls reward consumption, learning, memory, and addictive behavior (Polter and Kauer, 2014; Morales and Margolis, 2017). The VTA also has close connections to various brain regions whose input and output projections constitute a complex network of behavioral relationships in the VTA (Figure 1).

**Figure 2:**
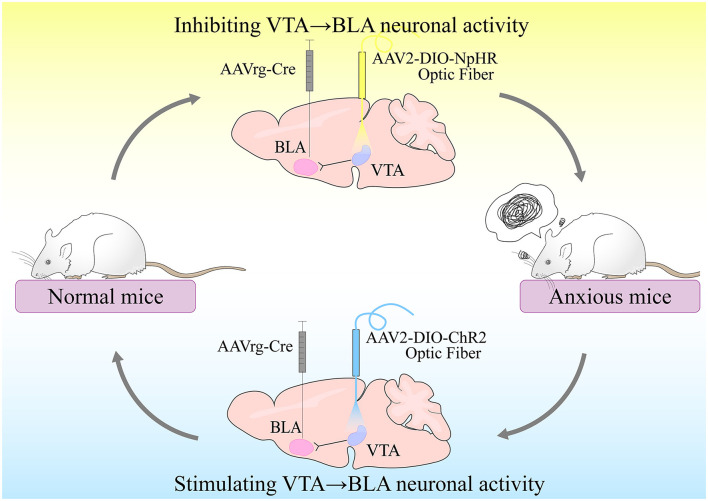
VTA-BLA neuronal activity controls anxiety-like behavior. Inhibition of VTA-BLA neuronal activity could produce anxiety-like behavior; activation of VTA-BLA neuronal activity could produce anxiolytic effects.

Morel et al. (2022) conducted experiments based on the preceding research to investigate the relationship between VTA-BLA neurons and anxiety disorders (Morel et al., 2022). The chronic social failure stress paradigm (CSDS) was used to first induce different phenotypes in C57BL/6J mice in this study. Following CSDS, mice of various phenotypes were subjected to depression-related social interaction test (SI), female urine sniffing test (FUST), and sucrose preference test (SP), as well as anxiety-related elevated plus maze (EPM) or open field test (OFT). They discovered that time spent in EPM open arms was related to time spent in the center of the open field using correlation data analysis. Time spent in EPM open arms, on the other hand, was not clearly related to social avoidance behavior. Female urine and sucrose preferences were also related to SI behavior but not to anxiety-like behavior test timing. It demonstrated that anxiety-like behavior was independent of depression-like behavior. The researchers then used a dual viral strategy to selectively label VTA projections to NAc and BLA neurons and counted the number of co-labeled neurons (only 2.7%). It was also shown that anxiety-like behavior was independent of depression-like behavior. Furthermore, VTA-BLA dopamine neurons in anxious mice had lower excitability, higher rheobase, I_h_ current, and sag amplitudes following whole-cell patch-clamp recordings and a video-tracking system synchronized with the fiber photometry system. All of these findings indicate that VTA-BLA dopamine neuronal activity is linked to anxiety-like behavior. Finally, Morel et al. (2022) used optogenetics to control the activity of VTA-BLA neurons selectively. The mice were found to have less time in the EPM open arm and the OFT center when subjected to sub-threshold social defeat stress (Sub.D) by using NpHR-optogenetic means to inhibit the activity of VTA-BLA neurons. In contrast, the mice increased their time in the EPM open arm and in the OFT center by using ChR2-optogenetic means to stimulate the activity of VTA-BLA neurons. As a result, Morel et al. discovered that VTA-BLA neurons regulate anxious behavior (Figure 2).

The study challenged the previously held belief that anxiety and depression share neural circuits and investigated the link between anxiety-like behavior and the midbrain dopamine system, which not only improved understanding of the midbrain dopamine system but also provided practical experimental thinking to investigate neural circuits. The experimental procedure of this study is impressive, but we still wish to discuss some details. First, NAc could not only receive neural projections from BLA, but it could also induce changes in anxiety behavior in previous studies (Zhang et al., 2020; Huang et al., 2021; Khastkhodaei et al., 2021). Sun et al. discovered that the VTA-BLA-NAc neural circuit regulates reward effects and motivated behavior (Sun et al., 2021). So, whether the presence of 2.7% of co-labeled neurons could indicate that anxiety-like behavior was not completely independent of depression-like behavior. Second, the VTA is a heterogeneous structure in which GABAergic and glutamatergic neurons modulate DA neurons (Gordon-Fennell and Stuber, 2021; Miranda-Barrientos et al., 2021). GABA neurons in the VP, for example, can be projected into VTA, which acts on DA neurons and modulates motivation; glutamatergic neurons in the LHb can also be projected into VTA, which inhibits DA neurons and modulates reward (Omelchenko et al., 2009; Hjelmstad et al., 2013). Whether the authors' electrophysiological experiments require them to differentiate DA neurons and selectively inhibit peripheral neurons. Finally, the authors did not explore the role of VTA-NAc dopaminergic neurons in anxiety (Nguyen et al., 2021). Whether adding control experiments can highlight the specific regulation of anxiety by VTA-BLA and make the experiment more complete. Whether the use of separate retrograde tracers in the same slice to record different dopaminergic neurons in VTA-NAC and VTA-BLA, would allow for more standardization of the experimental procedure.

Anxiety disorders have previously been linked to differential methylation of specific genes (MAOA, CRHR1, OXTR) (Schartner et al., 2017), and functional magnetic resonance imaging has revealed that the temporal and prefrontal regions of the brain respond differently in patients with anxiety disorders (Marin et al., 2020). In the treatment of anxiety disorders, the use of selective serotonin reuptake inhibitors (SSRI), selective serotonin norepinephrine reuptake inhibitors (SNRI), buspirone, benzodiazepines, and other drugs, in conjunction with psychotherapy that can enhance the effect of antidepressants (Strawn et al., 2018), has yielded positive results. However, the use of anti-anxiety medications causes many side effects in patients with anxiety disorders, such as allergies, headaches, gastrointestinal disorders, and so on (Balon and Starcevic, 2020; Panayotis et al., 2021). Furthermore, when selecting a drug, clinicians must consider a number of factors, including the patient's age, comorbidities, and tolerability (Katzman et al., 2014). Thanks to the efforts of Carole Morel and others, we have turned our perspective to the neuroscience research based on VTA-BLA, which will be the crucial part for the development of new anti-anxiety drugs. Anxiety is a common and non-negligible psychiatric symptom of the world's two most common neurodegenerative diseases, Alzheimer's disease (AD) and Parkinson's disease (PD) (Schrag and Taddei, 2017; Mendez, 2021). As a result, this research provides new hope for improving the quality of life of people suffering from neurodegenerative diseases.

## Discussion

The co-morbidity of anxiety and depression presents a clinical treatment and diagnostic challenge that has plagued countless patients and physicians (Choi et al., 2020). By demonstrating that VTA-BLA induces anxiety-like behavior via optogenetics, this study provided new insights into anxiety and depression and inspired the development of novel anxiolytic drugs. It also allowed us to complement further the neurobehavioral network associated with the VTA. In 2021, Nguyen et al. discovered that activation of VTA-amygdala DA neurons blocked the anxiety effects of nicotine (Nguyen et al., 2021); however, Jiang et al. (2021) discovered that activating corticotrophin-releasing hormone-mediated CeA-VTA terminals increased opioid withdrawal-induced anxiety and inhibiting CeA-VTA decreased anxiety. That is the inverse direction of the VTA-BLA regulation of anxiety. Because BLA and CeA are two critical functional regions in the amygdala, we wondered whether the anxiety-related VTA-BLA-CeA closed neural loop exists and what kind of connection exists between BLA and CeA.

Anxiety is a non-motor symptom that is common in AD and PD. Krashia et al. (2022) have recently proposed the VTA dopamine system as a therapeutic target for neuropsychiatric symptoms of AD. And PD is caused by the selective deletion of dopaminergic neurons in the dense part of the substantia nigra (SNc) (Tang et al., 2020; Zhang et al., 2021). Interestingly, Suzuki et al., found that a reduction in the number of VTA DA neurons was observed after unilateral injection of 6-hydroxydopamine (6-OHDA) in the midbrain of mice, and Alvarsson et al. (2016) found that overexpression of α-synuclein within the VTA after 3 weeks of adenovirus injection could lead to motor disability (Suzuki et al., 2010). It made us wonder whether VTA neural circuits are associated with the deficiency of SNc dopaminergic neurons and whether VTA could also be a new therapeutic target for PD.

## Author contributions

JZ, PS, and HL conceived the article. JZ and HL wrote the first draft and reviewed and revised the manuscript. All authors contributed to the article and approved the submitted version.
